# Managerial Areas of Construction and Demolition Waste: A Scientometric Review

**DOI:** 10.3390/ijerph15112350

**Published:** 2018-10-24

**Authors:** Jianguo Chen, Yangyue Su, Hongyun Si, Jindao Chen

**Affiliations:** School of Economics and Management, Tongji University, Shanghai 200092, China; jgchen@tongji.edu.cn (J.C.); chenjindao@tongji.edu.cn (J.C.)

**Keywords:** construction and demolition waste, CDW, management, scientometric analysis, review

## Abstract

In past decades, the massive generation of construction and demolition waste (CDW) was increasingly threatening the public environment and humanity health worldwide. A large amount of research has been devoted to the CDW from difference perspectives. However, few scholars have attempted to summarize and review the extant studies, especially in the managerial areas of CDW (MA-CDW). This paper fills this gap via a systematic and quantitative review in the CDW management field. Employing the scientometric analysis method, a total of 261 articles published from 2006 to 2018 were collected to construct the knowledge map and comprehensive framework for MA-CDW. Results show that the overall evolutionary trend of MA-CDW was from basic management concepts to internal and external challenges analysis, to organizational strategy and innovative management practices. The major MA-CDW knowledge domains were identified and summarized into four pillars, namely: (1) factor and challenge; (2) composition and quantification; (3) assessment and comparison; and (4) technology and method. Based on the trend, knowledge gaps and future research directions were found out and discussed. This study contributes to the existing MA-CDW knowledge by presenting a comprehensive knowledge framework. Furthermore, these findings can provide the researchers and practitioners with an in-depth understanding for the sustainable governance of CDW.

## 1. Introduction

Construction and demolition waste (CDW), the abandoned materials generated during construction, renovation, and demolition [1], is one of the heaviest and most voluminous waste streams produced globally [2]. CDW accounts for approximately 35% of all global waste [3], as well as 70%, 50%, 44%, 36%, and 30% of the total waste in Spain, United Kingdom, Australia, Japan and Italy, respectively [4]. Over the past few decades, inappropriate treatment and disposal of CDW have given rise to increasing environmental pollution, natural resource consumption, and land price, which places massive pressure and has negative impacts on human living environments [5]. In this context, the management areas of construction and demolition waste (MA-CDW) are gradually being given increasing global attention.

Numerous scholars have conducted research on the MA-CDW, using various research paradigms and methods, from different perspectives and disciplines. Yuan and colleagues focused on investigating the practices, challenges and strategies of CDW in China, especially in Hong Kong [6,7,8]. Tam and colleagues concentrated on comparing waste management performance in different countries as well as evaluating the environmental and economic benefit of waste measures and policies [9,10,11,12]. In addition to assessing the environmental sustainability of recycled aggregates [13,14], Poon and colleagues also analyzed the waste reduction potential of prefabrication [15,16]. However, few studies have attempted to summarize and review the existing research, especially in the MA-CDW.

While scholars such as Gálvez-Martos and Menegaki have reviewed the main research and best practice of CDW management [17,18], the former only examined CDW management in Europe, and the latter primarily focused on the factors, barriers and motivations affecting CDW management. In addition, Lu and Yuan developed a framework and identified the three common topics in the MA-CDW (i.e., waste generation, reduction, and recycling), in order to help readers understand CDW management research from 1996 to 2010 [19]. It should be noticed that the building of new infrastructure and housing construction in many countries has been accelerating since 2010. With the emergence of increasing CDW management challenges, a large number of new studies have been conducted and published in the past eight years. It is imperative to systematically examine the state-of-the-art advancements and emergent trends, in order to encourage future studies and innovative practices. Therefore, the main objectives of this study are: (1) to summarize MA–CDW studies from 2006 to 2018; (2) to understand the holistic research status and evolutionary trend from the perspective of published journal articles, document co-citation, keyword co-occurrence, cluster analysis and burst detection; and (3) to develop a comprehensive framework for MA-CDW, including major knowledge domains, gaps, and future directions. To achieve these research goals, we employ the scientometric analysis method, which is used to map the visualization review of a specific knowledge area. This paper provides valuable guidance and in-depth understanding for researchers, practitioner and policy makers to promote CDW sustainability. Furthermore, this study contributes to the existing body of knowledge of MA-CDW by presenting a new, integrated, and holistic knowledge framework.

## 2. Research Method and Processes

### 2.1. Data Collection

The Web of Science (WoS) core database covers the most prestigious and important journals all over the world, and thus is considered to be the most authoritative database for studying literature in many fields [20,21]. Although Scopus covers a wider range than WoS, there are many overlaps between the two databases. The Web of Science core database contains the most influential and prestigious publications on MA-CDW research, and is therefore selected as the data source for this paper.

Data for the contribution was extracted from the WoS Core collection database (SCI-EXPANDED, SSCI) in May 2018. In order to conduct a comprehensive analysis, the following steps have been followed. In the first stage, we go through a number of highly-cited publications on the topic of construction and demolition waste to identify the related key terms. Then, a combination of most frequently appearing search terms about CDW were selected after reviewing preliminary papers. Papers containing these search terms in the title, abstract and keywords were selected. The final search terms included “TS = (“construction waste *” OR “demolition waste *” OR “construction and demolition waste *” OR “CDW” OR “C&DW”) AND TS = (“management” OR “managerial” OR “managing” OR “manage”)”. The language of the publications was limited to English and document type was limited to articles, the time span was set to 1975–2018. As a result, 398 bibliographic records were retrieved.

The boundary of MA-CDW can be defined as reduction/minimization, reuse, recycling, generation, impact, regulatory environment, performance measurement, manpower, and tools assisting in CDW [19]. Based on this boundary, a manual review of paper titles and abstracts was conducted to exclude articles concerning CDW material performance, such as physical characteristics, mechanical properties, and micro-structural characteristics. After manual review, a total of 281 papers were selected.

**Figure 1:**
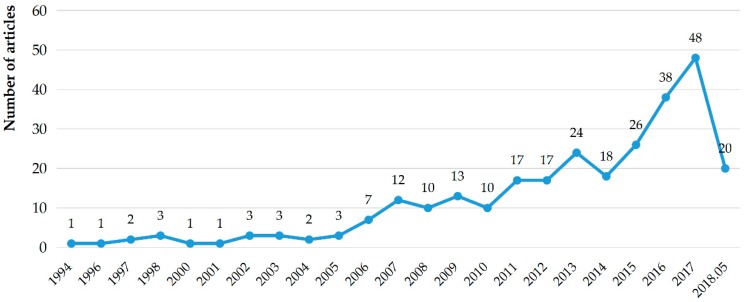
Number of studies on MA-CDW from 1994 through 05/2018.

Figure 1 displayed the time-trend analysis of 281 MA-CDW studies. The number of publications relating to CDW management increased significantly from 2006 to 2018. This is because many countries and regions introduced waste management legislation after 2006. For example, Hong Kong enacted a Waste Disposal Charging Scheme based on the polluter-pays principle in 2006 [22]; the UK introduced Waste Strategy for England in 2007, which set explicit CDW recovery targets in order to reduce the amount of waste going into landfills [23]; the EU enacted a Waste Framework Directive in 2008 pointing out the management of waste should not endanger human health and the environment [24]. These polices stimulated an increasing interest in MA-CDW research. Furthermore, with the concepts of sustainable development and circular economy being widely spread and accepted, the CDW governance research will continue to receive significant attention in the future. The subsequent analyzing process was thus set from 2006 to 2018 in CiteSpace. A total of 261 papers were ultimately selected for further analysis.

### 2.2. Scientometric Analysis

CDW management literature focuses on multiple disciplines; however, little attention is payed to characterizing the whole field through manual and scientometric review. Manual analysis tends to be subjective and limited in terms of the number of publications being reviewed as well as the relationship between publications cannot be analyzed [25]. Scientometrics analysis refers to application of mathematical and statistical methods to quantitatively analyzing the knowledge domain for a particular subject with large amount of articles [26]. Scientometrics analysis has been widely applied in architecture and construction research to investigate the characteristics, structure, hot topics and research trend (e.g., Ganbat et al. [27]; Li et al. [28]; Li et al. [29]; and Xue et al. [30]). It should be noted that the analysis process of scientometric analysis is quantitative, but the presentation of results can have qualitative characteristics.

Many scientometric analysis software have been developed in recent years, such as BibExcel, Ucinet, SCIMAT, VOSviewer, and CiteSpace. Some software (e.g., BibExcel, Ucinet) focuses on data processing, but they need to import the processing results into other visualization software (e.g., Pajek, Gephi) to display the network map [31,32]. SCIMAT, VOSviewer, and CiteSpace can not only process the data, but also visualize the results [33]. It is worth noting that these software assist researchers in scientific statistics and network mapping of literature, but cannot directly obtain summary results or conclusions. Comprehensive framework and network analysis require researchers to combine software output information with manual interpretation. Furthermore, the depth and quality of the interpretation are subjected to the researchers’ experience, knowledge, and academic background.

As a commonly used scientometric software, the tool applied in this study is CiteSpace, an automated scientometric analysis software for mapping and visualizing the intellectual structure of a scientific knowledge domain [34]. CiteSpace software provide several types of scientometric analysis, including collaboration network analysis, keyword co-occurrence analysis, author co-citation analysis, and document co-citation analysis [35].

The bibliographic record exported from WoS contains several elements, including cited times, authors, title, keywords, abstract, source journal, references and addresses. A large number of cited times commonly demonstrate that an article has high impact and the topic discussed in the article has received extensive attention. The title, keywords, and abstract can clearly explain the main content of the article. Therefore, four types scientometric analysis methods are used in this paper via CiteSpace. Document co-citation analysis is the first step to identify the knowledge domain by revealing the most cited publications. Co-word analysis is used to describe the hot topics by processing terms extracted from co-occurrences in titles, keywords, and abstracts [36]. Cluster analysis is conducted to discover the main cluster of the CDW knowledge domain. Keywords burst can identify emergent trends to facilitate knowledge evolution.

## 3. Research Results

### 3.1. The Major Published Journals

The 261 articles we extracted were published in 63 different journals. Table 1 shows the performance of top 15 most productive journals that have published at least three articles on MA-CDW from 2006 to 2018. The number and proportion of articles in the top 15 productive journals as well as journal impact are also shown in Table 1. The top 15 journals account for 78% of the 261 articles indicting that theses journals have a high concentration of articles on MA-CDW. Moreover, the top 4 journals account for 60% of the total articles (170 articles), which may imply that these four journals are the most renowned and influential publications on CDW management. *Resources Conservation and Recycling* published the highest quantity of articles with 55 (19.6%), much higher than the second and third journals, *Waste Management* 44 (15.7%), and *Journal of Cleaner Production* 36 (12.8%). It is also interesting to observe that the latter two journals (*Waste Management* and *Journal of Cleaner Production*) have a higher impact factor than *Resources Conservation and Recycling*. Lu and Yuan [19] also discovered 12 kinds of target journals related to MA-CDW through the WoS all database, ScienceDirect, EI Compendex and other database. Coincidentally, 10 of the 12 journals listed in their study are also included in Table 1 and the other two journals belong to the non-core database of WoS, which further confirms that the core set of WoS includes the most influential journals in the field.

### 3.2. Document Co-Citation Analysis

**Figure 2:**
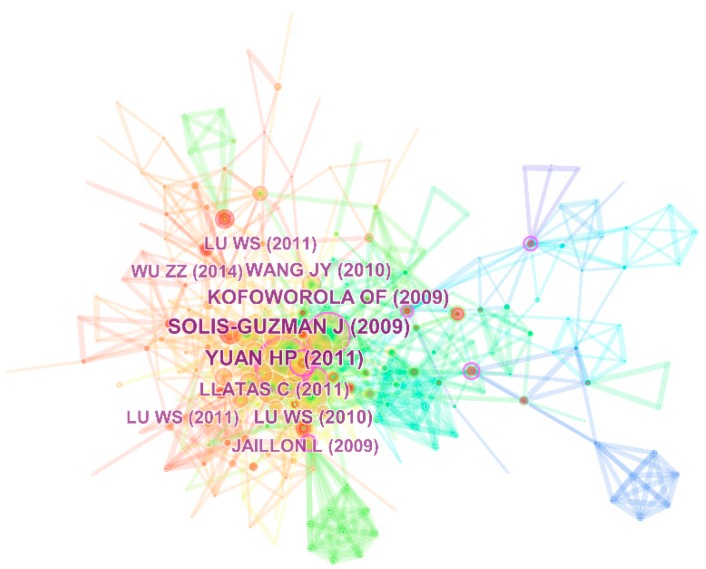
Document co-citation network for the MA-CDW: 2006–2018. Notes: Top 10 most cited documents were highlighted. For example, ‘LU WS (2011)’ in this figure represents detailed information of a document with high citation. ‘LU WS’ is name of the author and ‘2011’ is the published year.

Figure 2 shows the document co-citation network of MA-CDW studies generated by CiteSpace with 263 nodes and 963 edges. The time span of the data is from 2006 to 2018 and the time slice is set to one year (Figure 2). The top 50 most cited documents from each time slice are selected for generating the subsequent network. Nodes represent cited references and edges between two nodes represent co-citation relationships. Edge colors are consistent with the time slice at the top of the figure. The thickness of edges is proportional to closeness of the reference relationship, and the volume of nodes is proportional to the number of citations. The thicker the edge, the closer the relationship between the two references, and the larger the node, the more critical the study. Yuan and Shen [37] had the highest number citations, illustrating the research trend for the MA-CDW. This paper recognized the main authors to the MA-CDW research, main methods adopted by researchers and research trend of hot topics from 2000 to 2009. The second article [38] presented a model for quantifying CDW volume for new construction projects and demolition projects. As such, Kofoworola et al. [39] estimated the construction waste generation in Thailand from 2002 to 2005. Unlike Solís-Guzmán et al. [38], Kofoworola et al. further discussed potential feasibility and benefits of recycling construction waste based on the amounts and composition of construction waste.

For easy interpretation, the detailed information of top 10 most cited publications in Figure 2 are listed in Table 2. The top 10 articles were published from 2009 to 2014, and can be classified into 4 types, including literature review [19,37,40], quantification of CDW amount [3,38,39,41], impediment and strategy [42,43] and technology and method innovation in waste reduction [15]. Literature review articles being highly cited is not surprising as they illustrate the status and trends based on comprehensive and systematic analysis helping readers acquire in-depth understanding of a research field. Understanding the amounts and composition of CDW is a prerequisite for efficient management and prevention. Thus scholars aim to estimate the volume of CDW generation either at project level [44] or at regional level [39]. Critical factors and impediments for MA-CDW are analyzed to help stakeholders to develop effective CDW management strategies. It is noting that all of the articles are from the journal of Waste Management and Resources Conservation and Recycling, which are the top two journals in Table 1, indicating these two journals have good performance not only in the number of published articles but also in quality on the MA-CDW research.

### 3.3. Keyword Co-Occurrence Network

**Figure 3:**
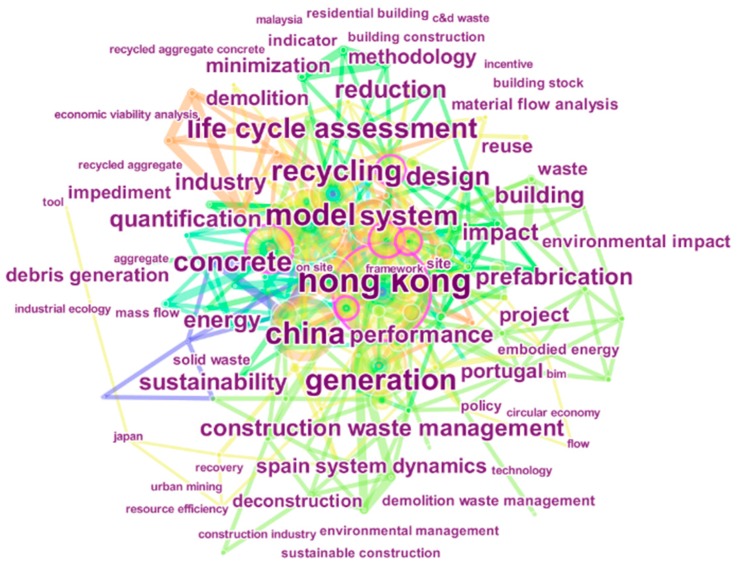
Keywords co-occurrence network in the MA-CDW: 2006–2018. Notes: “construction and demolition waste”, “construction waste”, “demolition waste” and “waste management” are treated as stop-words and are excluded in this figure.

Keywords denote the summary of primary content in studies and demonstrate the evolution of research themes over time. The keyword co-occurrence network is presented in Figure 3 and has 127 nodes and 492 edges. The timespan of the data is 2006 to 2018, and the time slice is set to two years. In this network map, nodes represent keywords that occur more than twice in the 261 publications selected in this paper. Edges connecting two keywords represent two keywords emerging in a single article. The size of keyword is proportional to its frequency. It should be pointed out that, “construction and demolition waste”, “construction waste”, “demolition waste”, and “waste management” are treated as stop-words. As they are the fundamental concepts of MA-CDW and they do not add substantial value to the present analysis. On the other hand, the frequency of these stop-words is much higher than other keywords, which will influence the interpretation of significant keywords in Figure 3 (the size of keyword is proportional to its frequency).

The most frequent co-occurrence keywords are “Hong Kong”, “model”, and “China” appearing 72, 53, and 47 times, respectively (Figure 3). This implies that CDW management has attracted extensive interests from scholars in China, especially in Hong Kong. Hong Kong has been committed to formulating construction waste management policy by applying the latest principles (e.g., 3R principles and polluter pays principle). These policies are relatively effective in regulating CDW management [45]. “Model” is the second highest ranking term, which are consistent with the study conducted by Yuan and Shen [37], which predicted that unlike former studies focusing on simple descriptive and statistical analysis methods, an increasing number of researches employ modeling technology (i.e., system dynamics, genetic algorithms, and integer programming model) to address complicated issues in the discipline of waste management.

The following high-frequent keywords “recycling” (frequency = 43), “generation” (40), “system” (36), “life cycle assessment” (34), “reduction” (23), “reuse” (12), and “impediment” (6) represent the hot topics in CDW management research, corresponding to the followed categories: (a) waste recycling, (b) estimation of waste generation, (c) waste management system, (d) life cycle assessment, (e) waste reduction, (f) waste reuse, and (g) impediment for waste management. As a widely accepted strategy for CDW minimization, waste recycling, reduction, and reuse are simultaneously included the categories. The topic of waste recycling has attracted the most research efforts. The category estimation of waste generation also received considerable interest from researchers because it is significant for government officials and practitioners to properly plan and control its disposal.

### 3.4. Cluster Analysis

**Figure 4:**
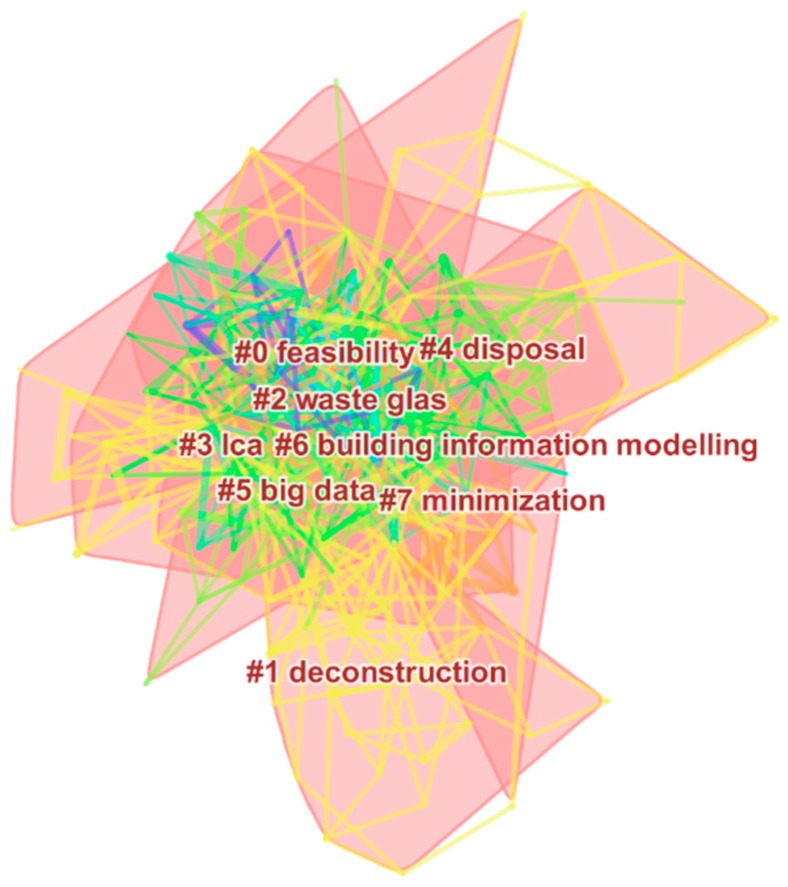
Cluster analysis in the MA-CDW: 2006–2018.

Keyword co-occurrence analysis has enabled us to grasp the hot topics in the field of CDW management, but word frequency cannot show the classification and knowledge structure of the field. Cluster analysis utilizes a series of algorithms to transform collected data into several structured clusters, thus discovering research patterns in the knowledge domain. In this paper, cluster labels are generated automatically by CiteSpace, which selects the top-ranked words occurring in each cluster as labels. Eight clusters are identified, such as feasibility, deconstruction, waste glass, LCA, disposal, big data, building information modeling, and minimization (Figure 4). The clusters modularity is 0.4878 (modularity > 0.3), and silhouette is 0.5308 (silhouette > 0.5), indicating the structure obtained by clustering is significant, and the result is robust and credible.

Further detailed information of eight research clusters are displayed in Table 3 in rank order, including cluster size, silhouette, and representative keywords. Cluster size represents the number of publications in each cluster, and silhouette signifies the homogeneity of a certain cluster. Silhouette values range from zero-to-one, and the closer the Silhouette value is to one, the higher the consistency of clustering members.

The most significant cluster is cluster #0 feasibility, including 28 articles (Table 3). These publications refer to the economic feasibility analysis for operating recycling plants and designing reverse logistics network; Cluster #1 deconstruction is related to the pros and cons on carrying out selective demolition and deconstruction; Cluster #2 waste glass focuses on the environmental impact of reused or recycled waste glass; Cluster #3 LCA covers the issues that use life cycle thinking to evaluate the environmental performance of CDW; Cluster #4 disposal refers to experiment studies on how to minimize waste at the disposal stage. The research themes of cluster #5 and cluster #6 concentrate on the application of big data and BIM (Building Information Modeling) in improving waste management, respectively; and Cluster #7 minimization covers the issues of methodology, indicators and practice in achieving waste minimization.

A few clusters, such as deconstruction, big data and BIM are not be noted in previous reviews [19,37], indicating that emphasis has been on technology innovation and application in the last decade. By comparing the main research topics from the co-occurrence analysis and cluster analysis, we find that there are some overlaps between the two results, such as life cycle assessment and waste reduction. There are also some differences between them. For example, cluster analysis identifies some new technology and methods applied in the MA-CDW, including deconstruction, waste glass, big data, BIM; whereas co-occurrence analysis reveals hot topics, such as waste recycling, estimation of waste generation and waste management system. These two results complement each other and enrich the understanding of MA-CDW.

### 3.5. Burst Detection and a Timezone View of Keyword

**Figure 5:**
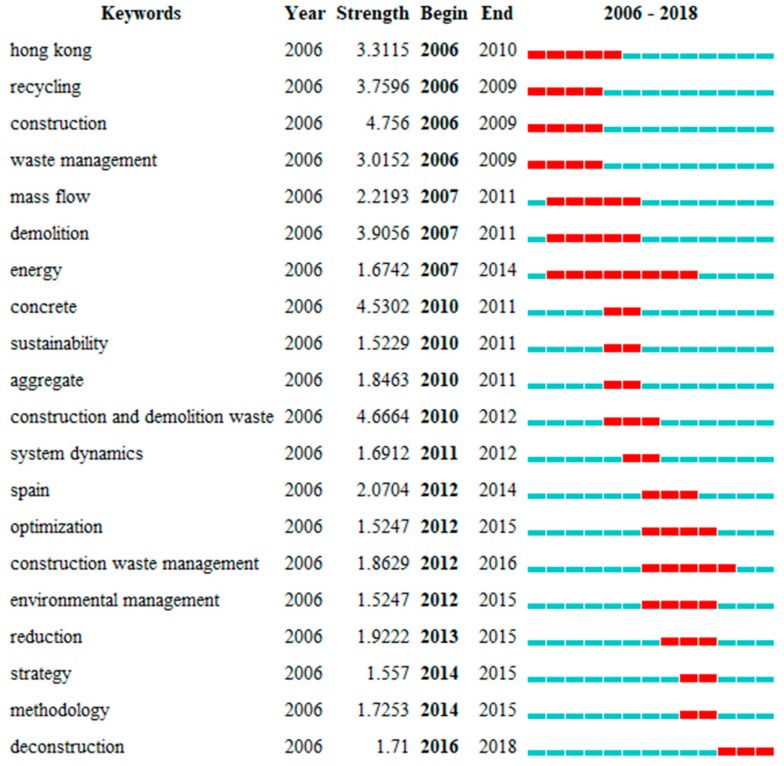
Top 20 keywords with the strongest citation bursts from 2006–2018.

Keyword co-occurrence analysis and cluster analysis enable us to understand the knowledge domain of CDW. However, changes in keyword frequencies through time have not been displayed. Burst detection is a tool that can detect the frequency and significant fluctuations of specific keywords in a short period of time. If the frequency of a keyword shows a dramatic increase in a short period, we can speculate that this keyword signifies an active sub-field. It should be noted that the keywords identified by burst detection may not be the word with the highest frequency. CiteSpace provides a keyword burst function to identify significant fluctuations over time as well as emergent trends in a knowledge domain [46]. Figure 5 shows the top 20 keywords with the strongest citation bursts in the CDW field from 2006 to 2018 in chronological order.

Research keywords from 2006 to 2009 have a burst time of at least four years, and the typical keywords in this period are “recycling”, “energy”, and “Hong Kong” (Figure 5). In this period, these studies focus on recycling practice in different economies [47], barriers or factors for waste recycling or on-site sorting [48,49], economic feasibility of recycling facilities [50], energy recovery in waste incineration, and energy consumption in environmental impact evaluation [51]. The frequency of the term “Hong Kong” experienced a significant surge from 2006 to 2010, this is likely related to the implementation of a Waste Disposal Charging Scheme by the Hong Kong government in 2006. After 2010, a diverse group of topics were presented by researchers and each topic lasted 2 to 3 years. As sustainable development gained worldwide attention, recycled concrete aggregate, the main product recycled from construction waste have surged from 2010 to 2011. Subsequently, numerous scholars engaged in “reduction” and “strategy”, including simulation of potential impact of policies and waste management approaches (i.e., recycling, landfill and incineration), waste minimization design, and elimination of illegal dumping [52,53,54].

**Figure 6:**
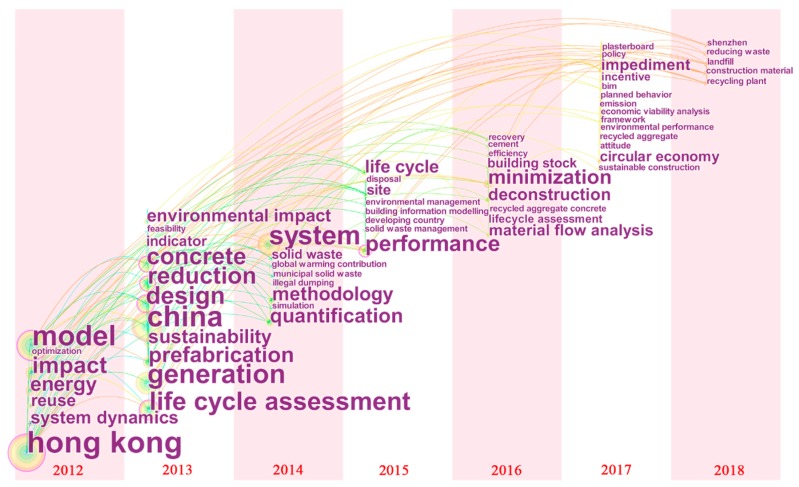
A timezone view of keywords occurring more than twice: 2012–2018.

Keywords burst detection enables us to comprehend holistic changes in the knowledge base since 2006, but detailed information on research hot topics in the past six years remains unclear. In order to further observe the research hot spots and trends of the last six years, a timezone view was constructed to present the keywords co-occurrence from 2012 to 2018 (Figure 6). The combination of timezone view and burst detection can better illustrate the evolutionary trend of a research field [55]. The links connecting nodes means co-occurrence relationships between keywords. The colors of these links are used to demonstrate when two keywords are connected. The sizes of node labels (keywords) are proportional with the occurring frequency of keywords.

Many keywords bursting from 2012 to 2018 (Figure 5), also appeared in keywords timezone view (Figure 6), such as system dynamics (2012), optimization (2012), reduction (2013), environmental management (2015), and deconstruction (2016), which reflect the main research focus in recent years. In 2012, two critical modeling approaches, system dynamics and optimization were widely adopted by scholars. The former was developed to understand the interaction between management measures and waste reduction effects from a dynamic perspective [56]. The latter involved reverse logistics network design from a regional CDW management perspective [57]. From 2013 to 2016, waste reduction became a hot topic, especially through design, prefabrication, site management and BIM measures. Similarly, quantify waste generation and building stock were also major topics from 2013 to 2016 [54,58,59,60]. It is noticed that BIM appeared in 2015 and 2017 with relatively low frequency compared to other keywords. However, this technology will be promising for enhancing the life cycle management of CDW by integrating information in the design, construction, maintenance, and demolition stages, as well as enhancing cooperation among stakeholders [61].

The timezone view from 2017 to 2018 may shows an emphasis away from a linear economy to a circular economy, because the frequency of “circular economy” outweigh that of “economic feasibility analysis” and “environmental performance”. In contrast to a linear economy, a circular economy emphasizes a closed-loop. As the circular economy is in its infancy in the field of CDW management, current research predominantly focuses on barriers, policy, and economic incentives to transition to a circular economy [62,63,64]. In addition, the planned behavior theory is applied to investigate CDW reduction behavior and the attitude of practitioners, particularly contractors, contractor employees, and designers [65,66,67].

## 4. Comprehensive Framework for the MA-CDW

**Figure 7:**
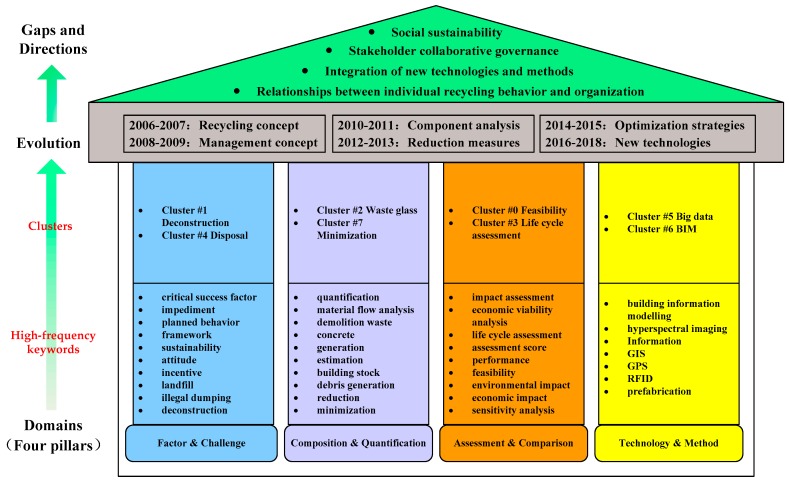
The comprehensive framework for MA-CDW: 2006–2018.

Systematic scientometric analysis provides the basic components to form an integrated framework for the MA-CDW. The comprehensive framework consists of three major parts: knowledge domains, knowledge evolution and potential future research directions (Figure 7). Based on the burst detection and a timezone view of keywords, the knowledge evolution in the MA-CDW has been illustrated in Section 3.5. In this section, we emphasize summarizing the main knowledge domains in the MA-CDW according to the results of keywords co-occurrence and cluster analysis. In addition, knowledge gaps are identified and some possible ideas that need further investigation are identified and discussed.

### 4.1. Knowledge Evolution

Based on the results of keyword detection and timezone view in Section 3.3, the evolution of MA-CDW can be detected. According to keyword detection, the major research topics from 2006 to 2009 were some basic concepts, such as “recycling”, “construction”, and “waste management”. These concepts could be regarded as the initial stage of MA-CDW development. Since 2010, themes changed to “concrete” and “aggregate”, implying that the research focus diverted from external challenges to internal component analysis (i.e., material). Publications from 2012 to 2013 focused on waste “reduction”, especially through “design” and “prefabrication” measures (Figure 6). From 2014 to 2015, “strategy” became the new concerns to prevent illegal dumping and enhance waste recycling. New technologies and methods such as “BIM” and “big data” became hot topics from 2016 to 2018. In a word, the evolutionary trend of MA-CDW from 2006 to 2018 can be summarized as transitioning from basic management concepts to internal and external challenges analysis to organizational strategy and innovative management practices.

### 4.2. Knowledge Domains

Based on the results of keywords co-occurrence and cluster analysis, knowledge domains in the MA-CDW are identified and further summarized into four pillars, namely, factor and challenge, composition and quantification, assessment and comparison, and technology and method. Detailed discussion and analysis are as follows.

#### 4.2.1. Factor & Challenge

Cluster #1 (deconstruction), Cluster #4 (disposal), and correlative high-frequency keywords constitute the first pillar (Factor and Challenge), which involves the factors and challenges that impede the management of CDW among various participants. Understanding the benefits, challenges, and processes of deconstruction is critical for successful implementation. Deconstruction and selective demolition are considered to be effective solutions for reducing demolition waste at source [68,69] and improving the waste recovery rate [70]; however, high labor cost, high technology demand, immature salvaged materials market, and other factors restrict its spread [71,72]. Construction waste disposal methods can be summarized into four types including on-site reusing, recycling, landfill, and illegal dumping [11], with landfill being the primary disposal option [73].

In addition, considerable research efforts have been devoted to the critical success factors and challenges at the industry level [42,64,74,75], project level [43,59,76,77,78], and individual level [65,66,79,80]. At the industry level, according to Jiménez-Rivero [74], more than half of the key factors are concerned with policy, especially regulatory instruments, which must be accompanied by economic incentives and other control strategies to achieve satisfactory results. It is worth noting that the research on an individual level, mainly based on the TPB (theory of planned behavior) framework explores the influence factors shaping waste disposal behavior. This theory pays considerable attention to the relationship between attitude and behavior in waste management [79]. Bakshan [65] found that the influence of personal factors such as attitude on behavior in CDW is more significant than that of other corporate factors such as training.

#### 4.2.2. Composition & Quantification

The second pillar Composition and Quantification includes Cluster #2 (waste glass), Cluster #7 (minimization), and correlative high-frequency keywords, which concern estimation and quantification of waste composition, waste generation rate and building stock. Acquiring accurate waste composition and generation data is a critical step for carrying out an effective waste management scheme and enhancing waste minimization. Waste quantification methodology can be divided into six types: site visit method, waste generation rate method, lifetime analysis method, classification accumulation method, variables modeling method, and other particular methods [40]. Each methodology has its application scope and conditions. It is difficult to say which method is most effective, because the different quantitative targets and different data that are collected determine the need for different methods under different conditions. It is noteworthy that the materials flow analysis method is commonly applied by researchers when estimating waste generation.

Currently, information technologies are commonly applied, and contribute to CDW volume quantification. Banias et al. [81] developed a web-based CDW quantification system and estimated 21 different waste streams for 4 types of buildings. Based on the research of Banias, Li and Zhang [82] further developed the web-based system to estimate construction waste, improving system accessibility, the interface, the connection, and information sharing. The accuracy of waste forecasting is highly dependent on the available data. BIM with all the information from the design stage to the demolition stage of the building has potential advantages for predicting the amount of CDW at the project level [83]. Through extracting material and volume information from BIM, it is possible to automatically estimate the waste generation not only from the construction stage but also from the demolition stage in the early design stage [84]. In addition, to accurately forecast the waste production based on building stock at the regional level, the geographic information system (GIS) presents as an innovative approach to assessing the amount of demolition waste [85] and monitoring the demolition activities [86] in space and time.

#### 4.2.3. Assessment & Comparison

Cluster #0 (feasibility), Cluster #3 (life cycle assessment) and correlative high-frequency keywords constitute the third pillar (assessment and comparison), which can be divided into two dimensions, economic feasibility analysis, and environmental impact assessment in different waste disposal scenarios (e.g., on-site reusing, recycling, landfill). Within the dimension of economic feasibility analysis, multiple scholars have examined the economic viability of CDW recycling plants [87,88,89,90,91], as well as economic viability of recycling programs [92,93]. According to studies about recycling plants, the results vary from high economically feasible [87], to feasible in certain circumstances such as charging gate fees [88], or extra revenue from location advantage [89], or installation of second-hand equipment [91] to not feasible [90]. Moreover, emerging studies are conducted from the perspective of a regional waste recycling network. For example, Fu proposed a reverse logistics network model based on the trade-off between cost and recycling rate, considering the location of facilities and best transport route [94]. Hiete presented a model that integrates CDW supply and recycling demand for minimum costs, concluding that disposal taxes are a cost-effective lever to enhance recycling [95]. In summary, economic viability is closely related to specific region and is influenced by physical, economic, and social factors [87].

In terms of environmental impact assessment, life cycle assessment (LCA) is a commonly used decision support tool in evaluating environmental impacts associated with the life cycle of products (goods or services) [96,97]. In the LCA approach, energy consumption and CO_2_ emission are the two most evaluated impact categories [98]. Application of the LCA method can be classified into six aspects: environmental impacts of a building [44,99,100], environmental impacts of construction waste in the construction phase [101,102], environmental impacts of demolition waste in the end-of-life phase [103,104], environmental impacts of demolition waste in the refurbishment phase [105], environmental impacts of recycled aggregates [106,107,108,109], and environmental impacts under different CDW management strategies [14,110,111,112,113]. Results show that, compared with landfill, most of waste recycling and reuse methods bring net environmental benefits [14,101,106,110]. On-site recycling environmental benefits are higher than off-site recycling [14,104,105,112], and the environmental benefits of off-site recycling are affected by transport distance [113].

#### 4.2.4. Technology & Method

The fourth pillar, technology and method, includes Cluster #5 (big data), Cluster #6 (BIM), and correlative high-frequency keywords, and centers on the adoption of information technology (i.e., big data, BIM, GIS, and RFID) and methodology (i.e., prefabrication) in transforming traditional CDW management. The existing CDW management tools, such as waste management plan templates and guides, waste data collection and audit tools, waste quantification tools, and environmental impact assessment tools have the following problems, including insufficient data quality for waste management, inability to integrate with the design process and lack of interoperability with other software [61]. In view of the potential deviation caused by insufficient quality data, big data provides the possibility of achieving a more comprehensive scene [114]. For example, Lu compared the construction waste management performance between public and private sectors through big data [115]. Furthermore, Chen recognized factors influencing demolition waste generation by connecting several databases [116].

GIS has advantages in data acquisition, storage, correlation, processing, and analysis. In addition to estimating generation of demolition waste, GIS can build a bottom-up material stock model which integrates with the LCA to assess the environmental impact at the urban scale [103] and integrates with GPS technology to provide real-time location of the material and its arrival time to the construction site [117]. More recently, Seror and Portnov employed GIS to identify areas at potential risk of illegal CDW dumping [118]. RFID tags are another data collection technology that can be employed to track CDW movement. Zhang proposed a framework that combined Rule-based Reasoning technology and RFID technology to track, schedule, and intelligently handle incidents of waste movement [119]. BIM is one of the space technology and data communication technologies commonly used in the architecture, engineering, and construction (AEC) industries, and can be effectively integrated with identification and data acquisition technologies (i.e., GIS, RFID, GPS). Integrating these technologies into BIM facilitates location-based management, tracking of building materials, and remote data collection.

### 4.3. Knowledge Gaps and Future Research Directions

According to the review of CDW management research from 2006 to 2018, this paper aims to determine gaps and some potential ideas that need further investigation. The gaps and future research directions are listed as follows.

The first knowledge gap is to seek stakeholder participation and collaborative governance in the life cycle of construction project. Collaborative governance is regarded as the best management model for various practices in public administration, which means that the public sector, the private sector, and other stakeholders participate in collective forums for consensus-oriented decision making [120]. The governance of CDW recycling is extremely complex, as it involves a large number of stakeholders from different sectors throughout the construction process. While existing research has largely investigated the recycling of CDW from a specific participant’s perspective, more research is needed to explore how to promote multi-sectoral participation and collaborative governance.

The second knowledge gap is to explore the relationships between individual recycling behavior and organization (i.e., construction company, design company). Since individual behavior is influenced by the interaction between the organization and the environment, studying the psychology and behavioral rules of individuals in a particular organization may improve the ability of managers to predict and guide the behavior of workers, thus achieving the goals of the organization more effectively. While some studies have focused on surveying behavior and attitudes that contribute to waste reduction, most studies operate from an individual behavioral perspective [67,80]. As the body of construction waste generation and recycling, how the organization interferes with the individual’s recycling behavior, and how the individual is affected (e.g., through performance assessing) are significant issues that require further exploration.

The third knowledge gap is the inadequate attention to social sustainability in assessing CDW treatment strategies. It is widely acknowledged that sustainability analysis should include the assessment of the environmental, social, and economic impacts. However, existing studies have only focused on the economic and environmental aspects of recycling CDW, while overlooking the social sustainability. Therefore, future studies are suggested to develop a social sustainability assessment method, including framework, categories, indicators, system boundaries, and impact assessment index. In addition, more research is desired to consider the economic, environmental, and social indicators simultaneously to draw more comprehensive conclusions.

The fourth knowledge gap is the integration of new technologies and methods. With the rapid development of information technology, more and more new technologies and methods are applied to construction projects, such as BIM, RFID, GIS, GPS, and big data. For instance, Kim proposed a BIM-based method that calculates demolition waste in the design phase [84]. Akinade developed a BIM-based model to determine the deconstruct ability in the design stage [121]. As such, future studies can extend the application of BIM from the design stage to construction, maintenance, and end-of-life stage, and simultaneously extend the BIM functions from waste generation estimation to waste management cooperation among stakeholders and waste analysis throughout a building’s life cycle. Moreover, integrating other data collection and data processing technologies (i.e., big data, GIS and RFID) into BIM is also necessary.

In addition to the gaps described above, close-loop material recycling of CDW need to be further studied in the context of a circular economy. In other words, the materials and components at the end of their life should be reused or recycled as resource in the future life cycles other than disposed as waste to landfill [122]. With respect to regional construction and demolition waste management, the reverse logistics network design with uncertainties from multiple objectives (i.e., economic, environmental, and social benefits) or parameters (i.e., supply, demand, cost, distance, waste quality, and recycling rate) will also be a significant direction.

## 5. Limitation

This work revealed the knowledge evolution, knowledge domain, and knowledge gaps about MA-CDW. However, with regard to the research method, three limitations need to be acknowledged. The first limitation of this paper is that the publications used for scientometric analysis were only collected from Web of Science core database and those from non-core databases were excluded. This may lead to insufficient results. The next limitation is that this paper only focused on the quantitative assessment of how often different topics have emerged in the MA-CDW, which were used to indicate the hot issues in this study. However, the occurring frequency of a topic may fail to show the importance of the topic. The last one is that some results such as the number of citation, number of co-occurrence will be changed at different searching time with the updating of databases. Future studies can extract publications from the latest databases to obtain the newest results.

## 6. Conclusions

This study systematically reviewed MA-CDW publications from 2006 to 2018 by using the scientometric analysis method. A total of 261 papers were selected for co-citation analysis, keyword co-occurrence, cluster analysis, and burst detection, in order to provide a holistic knowledge summary of the MA-CDW.

*Resources Conservation and Recycling, Waste Management, Journal of Cleaner Production,* and *Waste Management* & *Research* were identified as the four major journals associated with research on the MA-CDW. Yuan and Shen (2011), Solís-Guzmán et al. (2009), Kofoworola et al. (2009), Lu and Yuan (2010), and Llatas (2011) were recognized as the five most critical articles. By measuring the high-frequent co-occurrence keywords, the major research topics in this area include “waste recycling”, “estimation of waste generation”, “waste management system”, “life cycle assessment”, “waste reduction”, “waste reuse”, and “impediment for waste management”. 

Based on scientometric analysis, this paper has further proposed a comprehensive framework for the MA-CDW, including knowledge evolution, knowledge domains, knowledge gaps, and potential research directions. The overall trends of MA-CDW from 2006 to 2018 were summarized as from basic management concepts to internal and external challenges analysis, organizational strategy, and innovative management practices. The main knowledge domains of MA-CDW were identified and further classified into four pillars, namely: (1) factor and challenge; (2) composition and quantification; (3) assessment and comparison; and (4) technology and method.

Based on the analyses of knowledge evolution and domains, knowledge gaps and future research directions were ultimately discussed as well. (1) Considering that most of the existing research investigated the recycling of CDW from a single participant’s perspective, more research is needed to explore multi-sectoral participation and collaborative governance. (2) The second knowledge gap is to examine the relationships between individual recycling behavior and organization (i.e., construction company, design company). (3) Current CDW life cycle assessment research mainly focuses on economic and environmental impacts, while neglecting the social impact. Future studies are recommended to develop a social sustainability assessment method for the CDW, which include framework, categories, indicators, system boundaries, and impact assessment. (4) The fourth knowledge gap is the integration of new technologies and methods. In the future, more work is needed to extend the application of BIM from the design stage to construction, maintenance, and end-of-life stage. Furthermore, it is essential to extend the BIM functions from waste generation estimation to waste management cooperation among stakeholders and waste analysis. In addition, further research is needed to explore how to integrate other data collection and data processing technologies into CDW management process. (5) With the widespread of the concept of circular economy, more research is desired to devise effective CDW management frameworks and strategies. (6) Reverse logistics network design with uncertainties from multiple objectives or parameters is also a significant direction.

This study contributes to the existing MA-CDW body of knowledge by constructing a comprehensive knowledge framework and providing current status, evolutionary trend, and future directions. These findings can help researchers and practitioners quickly understand MA-CDW research. In particular, knowledge domains and evolutionary trend can offer clear and in-deep cognition of MA-CDW research. The knowledge gaps point out some specific and urgent issues, as well as the research directions.

## Figures and Tables

**Table 1:** The performance of top 15 journals from 2006 to 2018.

No.	Journal Name	No. of Articles	%	Impact Factor
1	*Resources Conservation and Recycling*	55	19.6%	3.313
2	*Waste Management*	44	15.7%	4.03
3	*Journal of Cleaner Production*	36	12.8%	5.715
4	*Waste Management & Research*	27	9.6%	1.803
5	*Sustainability*	8	2.8%	1.789
6	*Building Research and Information*	7	2.5%	3.136
7	*Automation in Construction*	7	2.5%	2.919
8	*Building and Environment*	6	2.1%	4.053
9	*Journal of Industrial Ecology*	5	1.8%	4.123
10	*Engineering Construction and Architectural Management*	4	1.4%	1.613
11	*Environmental Engineering and Management Journal*	4	1.4%	1.096
12	*Journal of Construction Engineering and Management*	4	1.4%	1.735
13	*Waste and Biomass Valorization*	4	1.4%	1.337
14	*Europe Journal of Operational Research*	3	1.1%	3.297
15	*International Journal of Life Cycle Assessment*	3	1.1%	3.173

Impact factor: Journal Impact Factor (Clarivate Analytics) in 2017.

**Table 2:** The top ten critical publications in the MA-CDW.

Author	Title	Year	Cited	Journal
Yuan and Shen	Trend of the research on construction and demolition waste management	2011	49	*Waste Management*
Solís-Guzmán et al.	A Spanish model for quantification and management of construction waste	2009	47	*Waste Management*
Kofoworola et al.	Estimation of construction waste generation and management in Thailand	2009	37	*Waste Management*
Lu and Yuan	Exploring critical success factors for waste management in construction projects of China	2010	34	*Resources Conservation and Recycling*
Llatas	A model for quantifying construction waste in projects according to the European waste list	2011	31	*Waste Management*
Wang et al.	Critical success factors for on-site sorting of construction waste: A China study	2010	31	*Resources Conservation* *and* *Recycling*
Jaillon et al.	Quantifying the waste reduction potential of using prefabrication in building construction in Hong Kong	2009	27	*Waste Management*
Lu and Yuan	A framework for understanding waste management studies in construction	2011	26	*Waste Management*
Wu et al.	Quantifying construction and demolition waste: an analytical review	2014	24	*Waste Management*
Lu et al.	An empirical investigation of construction and demolition waste generation rates in Shenzhen city, South China	2011	24	*Waste Management*

**Table 3:** Eight research clusters in MA-CDW.

Cluster	Size	Silhouette	Top Keywords
#0 feasibility	28	0.551	benefit-cost analysis, recycling plant, recycling network
#1 deconstruction	26	0.703	challenge, technology, sustainability
#2 waste glass	21	0.643	recycled aggregate, environmental impact
#3 LCA	20	0.743	environmental benefit, energy consumption, CO_2_ emission
#4 disposal	20	0.653	waste disposal charging scheme, policy, polluter pays principle
#5 big data	16	0.661	performance, hyperspectral imaging, information
#6 BIM	15	0.633	integration, design, quantification
#7 minimization	11	0.782	methodology, best practice, design
